# Enhancing Diagnosis of Rotating Elements in Roll-to-Roll Manufacturing Systems through Feature Selection Approach Considering Overlapping Data Density and Distance Analysis

**DOI:** 10.3390/s23187857

**Published:** 2023-09-13

**Authors:** Haemi Lee, Yoonjae Lee, Minho Jo, Sanghoon Nam, Jeongdai Jo, Changwoo Lee

**Affiliations:** 1 Department of Mechanical Design and Production Engineering, Konkuk University, 120 Neungdong-ro, Gwangjin-gu, Seoul 05030, Republic of Korea; dlkgoal@konkuk.ac.kr (H.L.); dldbswp913@konkuk.ac.kr (Y.L.); als8080@konkuk.ac.kr (M.J.); 2 Department of Mechanical Engineering, Massachusetts Institute of Technology, Cambridge, MA 02139, USA; shnam@mit.edu; 3 Department of Printed Electronics, Korea Institute of Machinery and Materials, 156, Gajeongbuk-ro, Yuseong-gu, Daejeon 34103, Republic of Korea; micro@kimm.re.kr; 4 Department of Mechanical and Aerospace Engineering, Konkuk University, 120 Neungdong-ro, Gwangjin-gu, Seoul 05030, Republic of Korea

**Keywords:** feature selection, functional film, roll-to-roll manufacturing system, rotating element diagnosis, machine learning

## Abstract

Roll-to-roll manufacturing systems have been widely adopted for their cost-effectiveness, eco-friendliness, and mass-production capabilities, utilizing thin and flexible substrates. However, in these systems, defects in the rotating components such as the rollers and bearings can result in severe defects in the functional layers. Therefore, the development of an intelligent diagnostic model is crucial for effectively identifying these rotating component defects. In this study, a quantitative feature-selection method, feature partial density, to develop high-efficiency diagnostic models was proposed. The feature combinations extracted from the measured signals were evaluated based on the partial density, which is the density of the remaining data excluding the highest class in overlapping regions and the Mahalanobis distance by class to assess the classification performance of the models. The validity of the proposed algorithm was verified through the construction of ranked model groups and comparison with existing feature-selection methods. The high-ranking group selected by the algorithm outperformed the other groups in terms of training time, accuracy, and positive predictive value. Moreover, the top feature combination demonstrated superior performance across all indicators compared to existing methods.

## 1. Introduction

Roll-to-roll (R2R) manufacturing is an efficient production system that utilizes thin and flexible substrates, referred to as webs, to transport and process materials at high speeds using rolls and rollers [1,2]. This approach offers cost-effectiveness and environmental benefits [3]. Polymer-based webs, such as PET and PI, or metal-based webs, such as copper and aluminum, have gained widespread adoption in various fields, including flexible and wearable electronic products, perovskite-based solar cells, nanotechnology, and secondary batteries [4,5,6,7,8,9,10]. The performance of crucial rotating components for web transport, such as roll eccentricity and bearing defects, significantly affects the quality of the final products in R2R systems [11,12]. Malfunctions in these rotating components during web transport or winding can cause changes in web transfer speed and tension disturbances during processes such as printing and deposition, resulting in web deformations such as thickness and elongation variations [13]. In particular, the thin, flexible nature of polymer-based web and the increasingly thin metal film used for improved battery energy density make the web susceptible to deformation when tension disturbances occur [14]. This susceptibility to tension disturbances can lead to poor coating uniformity and significant defects within the functional layers, which can significantly impact the overall performance of the final product [15,16]. To enhance the coating quality of R2R systems, developing intelligent methodologies capable of monitoring, detecting, and diagnosing defects in rotating components that cause tension disturbances is crucial [17,18].

Prognostics and Health Management (PHM) is a technical research area that aims to minimize maintenance time by monitoring systems and detecting anomalies and failures [19,20]. Quality maintenance and fault diagnosis in R2R systems typically rely on sensors and inspection of the end product due to the behavior of the continuous web. Since it is difficult to inspect the workpiece in the field, developing an intelligent fault diagnosis system based on sensor data can reduce maintenance time [21].

Recent advances in technology have made it easier to collect massive amounts of sensor data, leading to an increase in sensor data-driven research [22,23,24,25]. As a result, researchers have accelerated the development of data-driven intelligent health-diagnostic models using machine learning and deep learning [26,27,28]. Machine learning methods and techniques generally follow a sequence of sensor-data collection, data-quality assessment, feature extraction, feature selection, and model training [29,30]. Data are collected from sensors attached to the machine, and vibration sensors have proven to be effective in diagnosing faults in rotating components in several studies [31,32,33,34]. The collected data are quantitatively evaluated for suitability in fault classification, and fault characteristics are quantified while selecting the optimal sensor [29,34]. As the measured signal contains noise, feature extraction is performed to extract only the information that reflects the state of the diagnosis target, excluding noise [35,36]. The extracted-feature set typically has a high dimensionality, and using all features as training data can reduce the classification accuracy and increase training time [37,38]. Therefore, performing feature selection is essential for choosing the most appropriate training data and quantitatively evaluating feature combinations that are relevant to faults [39,40]. Feature-selection engineering is an active research area, aiming to achieve benefits such as data reduction, training-time reduction, and enhanced accuracy [41,42,43,44].

Feature-selection methods can be divided into filter methods, wrapper methods, and embedded methods [45]. This study focuses on the filter methods that are fast to compute, can be combined with all kinds of prediction algorithms, and can be used for any high-dimensional datasets, as wrapper methods are not computationally able to deal with high-dimensional datasets and embedded methods are only used for certain algorithms [46]. However, the existing filter-based feature-selection method has a problem of lower accuracy at the expense of faster processing speed than other methods.

Therefore, we propose the feature partial density (FPD) algorithm, along with an accurate and quantitative evaluation method based on density- and distance-based classification effects using duplicate area data. We aim to achieve effective feature selection for fault classification and ensure accurate diagnostic performance. The core idea behind the FPD algorithm is to filter out the most valuable data by extracting the most relevant feature variable combinations from the sensor data. The FPD establishes a multidimensional-coordinate system by extracting feature combinations and calculates the partial density of areas based on feature variable sets. It then derives the FPD number (*FPD_n_*) by dividing the MD. Theoretically, the lowest *FPD_n_* indicates the lowest error data for the duplicate area. The diagnostic model constructed based on these data achieves the highest accuracy within the shortest training time.

To validate the effectiveness of our proposed algorithm, we conducted experiments using three-axis acceleration data collected from an R2R system for the diagnosis of roll eccentricity. We constructed an SVM [47] diagnostic model based on six high-rank cases, six low-rank cases, and six random extraction cases, using *FPD_n_*. We evaluated the performance of the model to validate the *FPD_n_*. Additionally, to diagnose bearings, which are an important element of rotation in roll-to-roll production systems, we constructed a fault diagnosis model using FPD and five existing filter-based feature selection algorithms based on Kaist rotor vibration data [48]. We compared the FPD with five existing methods for bearing diagnosis by building diagnostic models. We selected three commonly used filtering methods, mRMR, chi-square, and ReliefF, as well as MD evaluation and FDM methods that are highly relevant to the parameters used in this study. The superiority and generality of our proposed algorithm was confirmed through comparative evaluations.

## 2. Related Works

Feature selection reduces the large feature sets to the most significant features by minimizing the data’s dimensions. This step is critical for optimizing diagnostic efficiency with respect to predictive accuracy, learning time and storage needs [49]. Therefore, feature-selection research is considered one of the most productive and active fields of machine learning applications [50] with many feature-selection methods proposed in the last few decades [41,42].

Feature-selection methods can be divided into three main categories: filter methods, wrapper methods, and embedded methods, depending on whether or not they use a classification algorithm [45,51]. Filter methods rank features by calculating a score for each feature without using a classification model. In most filter methods, the score calculation is faster and more computationally efficient because it does not consume additional processing time by calling on a classification algorithm [52]. Wrapper methods, on the other hand, use a classification model to create all subsets and corresponding classification models for all features, and score each subset using the classification model’s performance measure. These methods can use optimization approaches such as metaheuristic algorithms [53,54]. Embedded methods combine the advantages of both methods including the feature selection for the model-fitting step [37,40].

In this paper, we focus on filter methods that are fast to compute, can be combined with all kinds of prediction algorithms, and can be used for any high-dimensional datasets, as wrapper methods are not computationally able to deal with high-dimensional datasets and embedded methods are only used for certain algorithms [46,53].

Li, Liang, Lin, Chen, and Liu [55] proposed a feature-selection method that uses multiple-scale form filters through the minimum redundancy maximum relevance (mRMR) [56] principle. To characterize and reduce data dimensionality, Dai, Xu, Wei, Ding, Xu, Zhang, and Zhang [57] developed an algorithm that considers the topology of data, thereby improving prediction performance. Uzun and Ballı [58] presented an algorithm that enhances classification performance by incorporating multivariate outliers and ReliefF feature selection. Koklu, Unlersen, Ozkan, Aslan and Sabanci [59] used the chi-squared test for feature selection and evaluated classifier performance using kernel support vector machine (SVM). Patel and Upadhyay [60] devised an algorithm for feature ranking in fault diagnosis by calculating the Euclidean distances between features. Suresh and Naidu [61] proposed a feature-selection method based on the analysis of variance (ANOVA) and Mahalanobis distance (MD) for SVM model-based multiple-class fault diagnosis. Lee et al. [29] introduced a quantitative feature-selection method that uses the feature-matrix volume and MD for diagnosing rotating machinery systems. Oh et al. [62] developed a feature selection method based on MD distance and a feature density matrix (FDM) for constructing a diagnostic model for the drive roll of an R2R slot-die coating system.

These filter-based feature-selection methods have demonstrated improvement, but there remains an opportunity to enhance accuracy. Therefore, we propose a new algorithm for feature selection which is determined by two parameters that are closely associated with model performance.

## 3. Theoretical Background

### 3.1. DNF Number-Based Data Evaluation

Directional nature of fault (DNF) is a technique to evaluate the quality of a dataset to quantify the condition or fault characteristics of measured data [36]. After collecting the sensor data through the experiments, the most effective dataset for fault diagnosis can be selected by evaluating the directionality of the faults for various sensor and axis data [31]. This method relies on the utilization of kurtosis and standard deviation as crucial measures. Kurtosis, being highly sensitive to impulses, is commonly employed for detecting faults in rotating elements [63]. Standard deviation, on the other hand, is utilized to evaluate the degree of imbalance in each signal [64]. The DNF number (*DNF_n_*) is defined in Equation (1), where α and β are weights between the kurtosis ratio and standard deviation ratio, $k_{n}$ and $k_{f}$ are the kurtosis derived from the normal and fault data, respectively, and $std_{n}$ and $std_{f}$ are the standard deviations of the normal and fault data, respectively. The highest *DNF_n_* value indicates the dataset that reflects faults most sensitively [31].

(1) $$DNF_{n}=\frac{1}{\alpha +\beta }\left(\alpha \frac{k_{f}}{k_{n}}+\beta \frac{std_{f}}{std_{n}}\right).$$

### 3.2. Feature Extraction

Feature extraction is the pre-process of extracting relevant and informative features from a given dataset, with the aim of capturing the inherent characteristics that reflect the underlying state of the diagnostic target [27]. By focusing on these pertinent features, feature extraction effectively eliminates noise and irrelevant information, enabling more precise and reliable data analysis [37]. Additionally, since feature extraction is a preliminary step to feature selection, improving the effectiveness can be achieved by extracting pertinent and important features in advance [65]. Following the filtration of the selected data using the DNF number, a compilation of significant industrial statistical features and time-domain statistical variables [66,67] were extracted, where X is the vector of vibration data, and N is a window size as listed in Table 1.

The combinations of feature variables were constructed from this extracted list of statistical feature variables. Each feature combination represents distinct cases that can be generated by employing different feature variables. Quantitative evaluation using the proposed algorithm enables the identification of optimal feature combinations from the constructed set, facilitating the development of optimal learning model data that excel in key metrics such as classification accuracy, positive predictive value (PPV), and learning time.

### 3.3. Mahalanobis Distance

MD is a statistical metric that measures the distance between information. It incorporates information from the covariance matrix, enabling a comprehensive assessment of distance. In classification, as the distance between classes increases, classification becomes easier, resulting in a reduction in misclassified data. In a multivariate space, MD is utilized to measure the distance between information. Unlike the more commonly used Euclidean distance, which solely considers the physical distance, MD considers the correlations between variables and provides a more accurate assessment of data distances [68]. Equation (2) can be used to calculate the MD between the class j data and sample data, where x represents the vector of class j data, m the vector of the mean values of the sample data, and C the covariance of the sample data.

(2) $$\;MD_{j}=\sqrt{{\left(x-m\right)}^{T}C^{-1}\left(x-m\right)}.$$

## 4. Material and Methods

### 4.1. Experimental Setup and Data Collection

**Figure 1:**
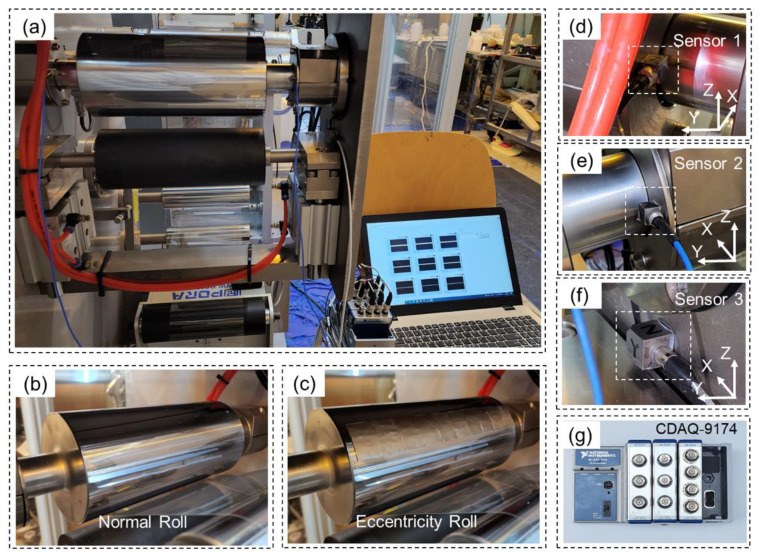
Experimental design: (**a**) R2R system and measuring equipment; (**b**) normal in-feeder roll; (**c**) eccentricity in-feeder roll; positions of sensors (**d**) 1; (**e**) 2; (**f**) 3; (**g**) CDAQ-9174 National Instruments board.

Figure 1 illustrates the experimental setup employed to validate the proposed algorithm. In this study, we assessed the effectiveness of the FPD algorithm using an industrial R2R system (Konkuk University), as depicted in Figure 1a. To evaluate the performance of the eccentricity diagnosis model, we introduced an eccentricity Figure 1c on an in-feeder roller Figure 1b of the R2R system. Three acceleration sensors Figure 1d–f were affixed to the roller to capture vibration data, which were acquired using a data acquisition (DAQ) board Figure 1g. To create the eccentricity, we cut a steel plate with a density of 7.5 kg/cm^3^ to dimensions of 20 mm × 30 mm × 0.5 mm and bent it to match the curvature of the roller. Subsequently, we applied eccentricity to the in-feeder roller and conducted an experiment using a PET film (CD901, Kolon Inc., Seoul, Republic of Korea). We collected all sensor outputs at a sampling rate of 12.8 kHz using data acquisition modules (DAQ NI-9230 and DAQ NI-9234) and LabVIEW 2018 version software (National Instruments, Austin, TX, USA). This experiment was repeated 3 times for 60 s. The specifications of the acceleration sensors are outlined in Table 2.

As shown in Table 2, the operating conditions of the R2R system and specifications of the acceleration sensors are indicated, including web speed, operating tension, and substrate of the R2R system and types, model of sensors and the sampling rate and duration of acquisition and types of DAQ and DAQ module.

### 4.2. KAIST Rotating Element Vibration Data

The generality of the proposed algorithm was verified using data collected by the Center for Noise and Vibration Control Plus in the Korea Advanced Institute of Science and Technology (KAIST) (Jung, et al.) [48]. In this study, the vibration data were collected under 4 Nm load with rated rotational speed of 3010 RPM. The vibration signals were measured using a total of four accelerometers (PCB352C34 PCB Piezotronics, Depew, NY, USA), which were attached to two bearing housings denoted A and B in the x and y directions. The data were sampled at a rate of 25.6 kHz. The state of the bearing condition was classified into five classes: normal, inner race fault, outer race fault, misalignment fault, and unbalance fault.

### 4.3. Design of FPD-Based Classifier

**Figure 2:**
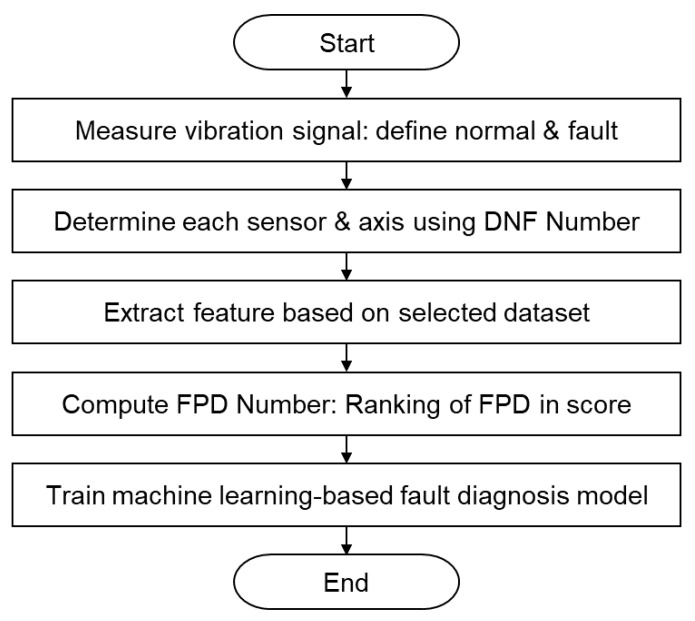
Flow chart of the fault diagnosis process with FPD methodology.

Figure 2 presents a flowchart outlining the process of designing a fault classifier using the proposed algorithm. The construction of the FPD-based classifier involved five distinct stages, which can be described as follows, when applying them to the experimental data for diagnosis of in-feeder roller eccentricity. Stage 1 encompassed the measurement of vibration data, acquired from an accelerometer sensor in the R2R system. Further details regarding this process can be found in Section 3.1. In Stage 2, the sensors and axes were selected based on the DNF number [28,33]. Specifically, the optimal dataset was determined by evaluating the DNF number for the nine datasets obtained from three sensors and three axes. Section 3.1 provides a detailed explanation of the methodology. In Stage 3, the feature combinations from the selected dataset were extracted. In this study, the chosen dataset was transformed into 20 statistical feature variables, and combinations of two different statistical feature variables were extracted. The list of the extracted statistical feature variables is presented in Section 3.2. Stage 4 involved the calculation and ranking of *FPD_n_* for the feature combinations. The efficiency of these combinations was evaluated using *FPD_n_*, enabling the selection of the most effective feature combination. Finally, in Stage 5, a machine learning model was constructed using the feature combination provided by the *FPD_n_* as the training data. For this study, diagnostic models were constructed using the top six, bottom six, and six random feature combinations identified by the *FPD_n_*. The performance of these models was then evaluated in terms of accuracy, training time, and PPV. Although this process was described for data collected for in-feeder roller diagnosis, it can also be applied to bearing diagnosis.

All diagnostic models were developed using MATLAB R2022a (MathWorks. Inc., Natick, MA, USA) and trained using the same computing power. The hardware used in the simulations is an Intel^®^ Core™ i9-11900F system (Intel Corporation, Santa Clara, CA, USA) with 16 GB of RAM, running on the Microsoft Windows 10 operating system.

### 4.4. Evaluation Method for Feature Combination Based on FPD Algorithm

**Figure 3:**
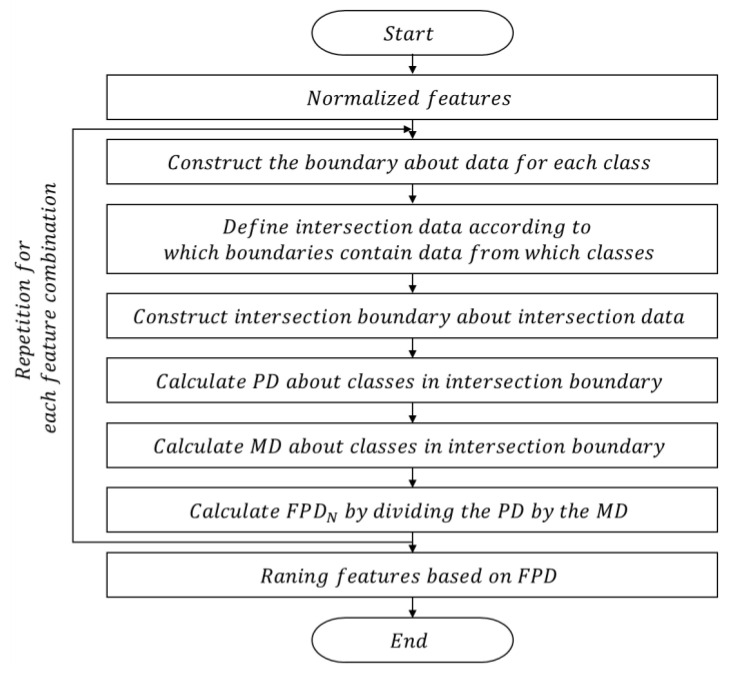
Flow chart of the feature selection methodology of FPD.

The flowcharts shown in Figure 3 provide a detailed and clear illustration of the FPD methodology, presenting the process of the FPD approach for quantitative feature selection.

First, as the method utilizes the distance between feature data, it is necessary to normalize the features beforehand for accurate evaluation and the building of a high-quality training dataset. Next, the boundaries constructed for each class of data and then the intersection data defined according to the class and location of the data. For example, if the data belong to class 1 and are within the boundary of class 2, they are deemed as intersection data. Likewise, if the data belong to class 2 and are within the boundary of class 1, they are also considered intersection data. Then, a boundary is constructed around the intersection data, and the PD and MD are calculated within the boundary. Subsequently, the FPDn is computed by dividing the MD by the PD. This process is repeated for other feature combinations, and once the FPDn is determined for all feature combinations, the optimal feature combination can be determined by ranking them according to FPDn magnitude.

**Figure 4:**
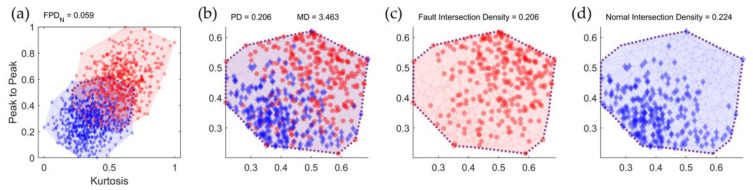
Comparison of distribution and distance in the intersection area of kurtosis–peak to peak: (**a**) data distribution over the entire area; (**b**) data distribution in the intersection area by class; (**c**) fault-data distribution in the intersection area (red); (**d**) normal-data distribution in the intersection area (blue).

When creating a feature-variable combination using two different types of feature variables, a two-dimensional plot can be generated, with each feature variable represented on an axis. In Figure 4, the blue data points represent healthy data, whereas the red data points represent defective data. Figure 4a illustrates the boundaries formed by connecting the outermost data points of each class, and the overlapping regions between the classes are defined as intersection areas, as depicted in Figure 4b. The overall intersection density is calculated by dividing the amount of data inside the intersection area by the total amount of data. Similarly, as shown in Figure 4c,d, the class-specific intersection density is determined by the ratio of the data within the intersection area to the total area of each class.

Data within the intersection area pose challenges in classification owing to the mixture of class data. Therefore, the classification accuracy tends to improve when the amount of data in the intersection area decreases compared to the total area, making intersection density a consideration for feature selection. However, because not all data within the intersection area are misclassified, the relationship between the overall intersection density and classification accuracy is non-linear. To enhance classification accuracy, it is necessary to consider the misclassified classes within the intersection area and adjust the density accordingly. The data belonging to the class with the highest density in the intersection region are classified correctly, whereas the remaining data, excluding these maximum density classes, represent classes that are likely to be misclassified within the intersection region. We define these remaining data as partial data. The sum of the intersection densities of the classes constituting the partial data is defined as the partial density (PD). As shown in Figure 4c,d, when the intersection density of the healthy class is lower than that of the defective class, PD is equal to the intersection density of the healthy class, as indicated in Figure 4b. If the total number of classes is n, and the class with the maximum intersection density is k, the PD can be expressed as shown in Equation (3).

(3) $$PD_{N}=\sum_{i=1}^{n}\frac{Number\;of\;intersection\;data_{i}}{Number\;of\;data}-\frac{Number\;of\;intersection\;data_{k}}{Number\;of\;data}.$$

There is an inverse relationship between the MD and classification difficulty for classes. Therefore, *FPD_n_* can be calculated by dividing the MD for other class data by the intersection density for each class, as shown in Equation (4).

(4) $$FPD_{n}=\sum_{i=1}^{n}\frac{Number\;of\;intersection\;data_{i}}{Number\;of\;data\times MD_{i}}-\frac{Number\;of\;intersection\;data_{k}}{Number\;of\;data\times MD_{k}}.$$

The FPD algorithm extracts intersection boundaries for each feature combination and evaluates the classification performance by considering the density of potentially misclassified class data and the MD within the intersection area. The feature combination with the lowest *FPD_n_* indicates minimal potential for misclassification and maximum MD for each class. Consequently, *FPD_n_* is calculated for each feature combination and they are sorted in ascending order to determine their ranking. A classification model built using high-ranking feature combinations may achieve superior classification performance, which encompasses accuracy, processing time, prediction speed, and PPV.

**Figure 5:**
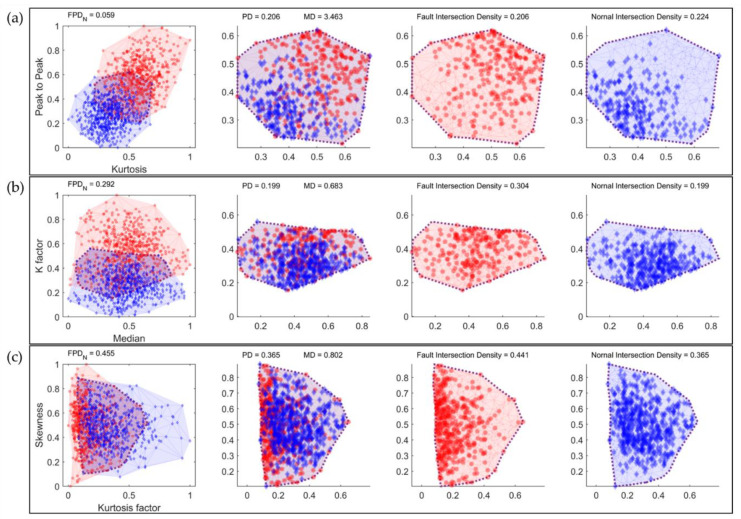
Comparison of distribution and intersection area according to feature combinations of normal and fault data: (**a**) kurtosis–peak to peak; (**b**) median K factor; (**c**) kurtosis-factor skewness.

Figure 5 illustrates the distributions of three feature combinations used to observe the effects of the PD and MD on *FPD_n_*. The model construction results for each feature combination are presented in Table 3. The kurtosis–peak to peak and median K factors exhibit similar PDs of 0.206 and 0.199, respectively. However, a significant difference exists in their MD values, with 3.464 for kurtosis–peak to peak and 0.683 for median K. The larger distance between the normal and defective data within the intersection for kurtosis–peak to peak suggests a better separation of the two classes, indicating easier classification.

However, the median K factor and kurtosis-factor skewness have similar MD values of 0.683 and 0.802, respectively, but notable differences in their PDs, which are 0.199 and 0.365, respectively. As the PD increases, the potential for data misclassification also increases, implying lower classification performance for kurtosis-factor skewness. In practice, model construction and diagnosis were conducted using each feature combination, and the results presented in Table 3 indicate that kurtosis–peak outperformed the median K factor in terms of training time, accuracy, and PPV. Furthermore, the kurtosis-factor skewness exhibited a decreased classification performance across all metrics compared to the median K factor.

### 4.5. Construction and Evaluation of Diagnostic Models Based on Selected Data

*FPD_n_* was calculated for all feature combinations, and a diagnostic model based on 5-fold cross-validation Gaussian kernel SVM was constructed using the top six high-ranked feature combinations, bottom six low-ranked feature combinations, and six randomly selected feature combinations. The performances of the constructed models were compared in terms of accuracy, training time, and PPV to assess the effectiveness of the number of FPDs.

Furthermore, the proposed feature-selection methods were validated by employing five representative or related feature-selection algorithms (mRMR, chi-square, ReliefF, MD evaluation, and FDM) to select feature combinations. Subsequently, a diagnostic model based on 5-fold cross-validation Gaussian kernel SVM was constructed using the selected feature combinations, and its performance was compared with the previous models in terms of accuracy, training time, and PPV.

## 5. Results and Discussion

### 5.1. Optimal Sensor Selection Based on DNF

In the R2R system, the in-feeder roller vibration data (IFR-V data) included sensor data from three sensors (sensor 1, sensor 2, and sensor 3) along with their X, Y, and Z axes, resulting in a total of nine datasets. Additionally, the KAIST bearing-vibration data (B-V data) included two sensor data for the X and Y directions for two housings (housing A, housing B), resulting in a total of four datasets. The datasets of IFR-V data and B-V data were evaluated using the DNF algorithm to determine their effectiveness. The evaluation results presented in Table 4 indicate that the Y-axis data from sensor 2 exhibited the highest DNF number about IFR-V data. Therefore, this dataset was deemed the most suitable for the diagnosis of eccentricity. Similarly, the evaluation results presented in Table 5 indicate that the Y-direction data from housing A exhibited the highest DNF number about B-V data. Therefore, this dataset was deemed the most suitable for the diagnosis of bearing.

The 20-feature variables shown in Table 1 were extracted from the dataset with the highest DNF number and 190 feature combinations, each consisting of two different variables, were constructed.

### 5.2. Eccentricity Diagnosis Results Based on the FPD Number for Each Feature Combination

**Figure 6:**
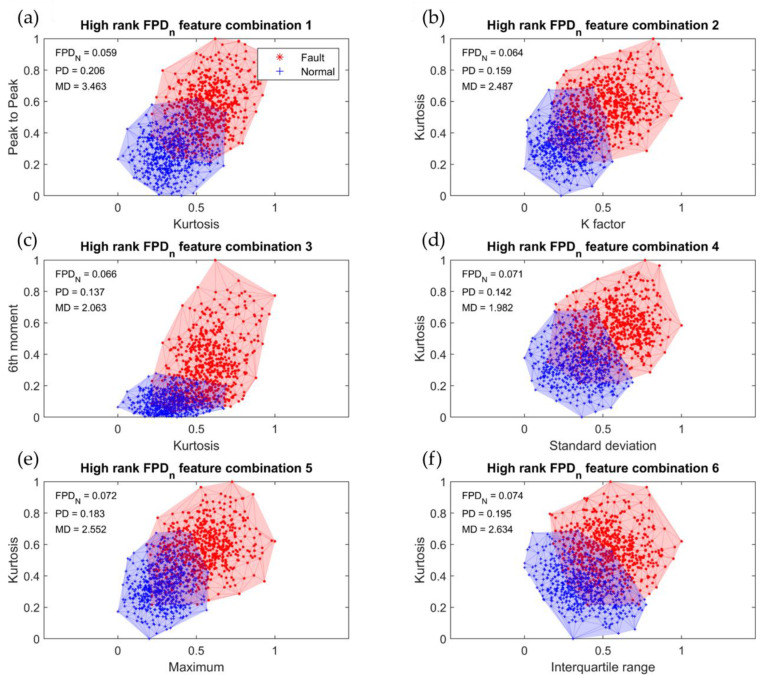
Six high-ranking feature combinations: (**a**) kurtosis–peak to peak; (**b**) K factor–kurtosis; (**c**) kurtosis–6th moment; (**d**) standard deviation–kurtosis; (**e**) maximum–kurtosis; (**f**) interquartile range–kurtosis.

Figure 6 displays a scatter plot showing the six high-ranking feature combinations obtained from the *FPD_n_* calculation on the IFR-V data. The red and blue data points represent the defect and normal classes, respectively. The corresponding *FPD_n_*, PD, and MD values for each feature combination are indicated in the upper left corner of the plot. The eccentricity diagnosis results of the R2R system-based classifier design, following the five steps outlined in Section 4.3, for the six high-ranking feature combinations are presented in Table 6. A higher *FPD_n_* value indicates a reduced overlap between class-dependent areas, indicating a better separation of data distribution for the normal and eccentricity cases and lower potential for misclassification. Additionally, owing to the significant distance between the class-dependent distributions in the overlapping areas, we anticipated a strong classification performance. The accuracy achieved using the six high-ranking feature combinations demonstrated excellent performance, ranging from a minimum of 89.08 to a maximum of 91.33%.

**Figure 7:**
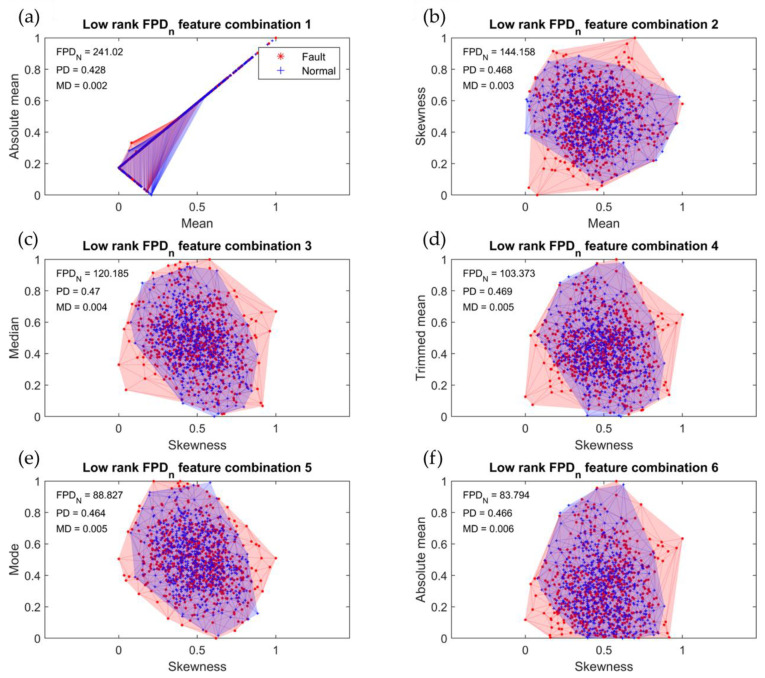
Six low-ranking feature combinations: (**a**) mean–absolute mean; (**b**) mean–skewness; (**c**) skewness–median; (**d**) skewness–trimmed mean; (**e**) skewness–mode; (**f**) skewness–absolute mean.

Figure 7 illustrates the six feature combinations with low rankings as determined by the *FPD_n_* output. The eccentricity diagnosis results of the R2R system, obtained through the application of the five-step algorithm-based classifier design proposed in Section 4.3, are presented in Table 7 for these low-ranking feature combinations. A low *FPD_n_* value suggests that the data distributions for normal and eccentric cases exhibit similarities, leading to overlapping areas between classes and a high density of misclassified data. Additionally, the distances between the distributions of each class within the overlapping areas were small, making accurate classification challenging. The accuracy achieved using the low-ranking feature combinations ranged from a minimum of 47.08 to a maximum of 54.42%, indicating significantly poor performance.

**Figure 8:**
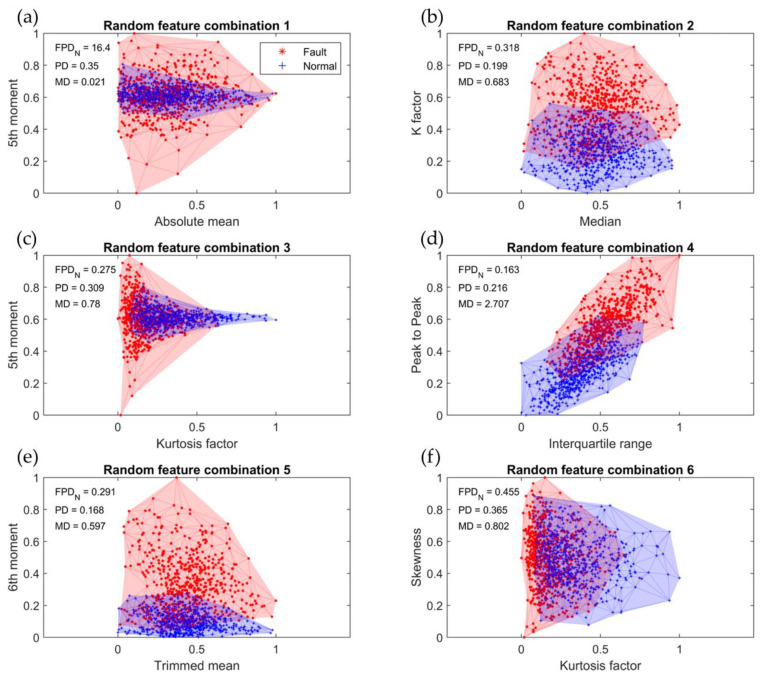
Random feature combinations: (**a**) absolute mean–5th moment; (**b**) median–K factor; (**c**) kurtosis factor–5th moment; (**d**) interquartile range–peak to peak; (**e**) trimmed mean–6th moment; (**f**) kurtosis–factor skewness.

Figure 8 depicts the six feature combinations that were randomly selected, and Table 8 displays the results of the eccentricity diagnosis for the R2R system obtained through the application of the five-step algorithm-based classifier design proposed in Section 4.3 on these randomly selected feature combinations. These random selections were made without using the FPD algorithm. The accuracy varied significantly, ranging from a minimum of 67.7 to a maximum of 89.2%, highlighting the substantial performance variation that arises when feature combinations are chosen randomly. Hence, employing a suitable algorithm for the selection of appropriate feature combinations is crucial.

Table 9 displays the average values of *FPD_n_* and the diagnostic indicators of machine state, such as training time, accuracy, and PPV, for the diagnostic models constructed using six high-ranked feature combinations, six low-ranked feature combinations, and six randomly selected feature combinations.

Comparing the results, the six high-ranked feature combinations exhibited a training time that was 30.25% lower than that of the six low-ranked combinations, along with an accuracy and PPV 37.90 and 38.32% higher, respectively. Additionally, when compared to the six randomly selected feature combinations, the six high-ranked feature combinations demonstrated a training time 18.75% lower, as well as an accuracy and PPV 10.11 and 5.39% higher, respectively. These findings highlight the close relationship between *FPD_n_* and the classification performance, confirming the appropriateness and effectiveness of feature combination selection based on the FPD algorithm in the development of models for eccentricity diagnosis in R2R systems.

### 5.3. Diagnosis of Bearing Fault via Comparison with Feature-Selection Algorithms Proposed in Prior Studies

Table 10 presents the machine learning performance metrics for the proposed algorithm and representative feature-selection methods (mRMR, chi-square, ReliefF, MD evaluation, and FDM) based on B-V data using kernel Gaussian SVM-based five-fold cross-validation. The metrics include accuracy, training time, and PPV. The *FPD_n_*-based classifiers demonstrated lower training times compared to those using other feature-selection algorithms (mRMR, chi-square, ReliefF, MD evaluation, and FDM) with reductions of 44.17, 53.03, 56.01, 57.29, and 15.56% respectively. Furthermore, the accuracy of the *FPD_n_*-based classifiers was higher, exhibiting improvements of 2.06, 8.45, 5.46, 11.53, and 0.83%, respectively, compared to that of other algorithms. Similarly, the PPVs of the *FPD_n_* -based classifiers were higher, with improvements of 1.67, 8.18, 5.49, 11.40, and 0.81%, respectively. In summary, the classifiers employing the proposed algorithm achieved lower training times than those using other feature-selection methods, with an average reduction of 44.17%. Moreover, the classification accuracy and PPV of the proposed algorithm were higher, with average improvements of 5.81 and 7.58%, respectively, compared to those of other algorithms.

The proposed algorithm demonstrates superior performance compared to other feature-selection algorithms in terms of training time, accuracy, and PPV. The reasons are as follows. The representative filtering feature-selection methods, MRMR, chi-square, ReliefF have the limitation of considering only independent statistical features and distributions. MD evaluation can reflect the correlation of two features based on the distance, but it has low accuracy because it does not introduce the density of the data, and FDM considers both density and MD together and achieves better results than other feature-selection techniques by reflecting the correlation of two features together. However, it does not achieve the highest accuracy because it does not introduce the partial density, which is the parameter most closely related to the misclassification rate. The proposed algorithm had the best performance because it selected the features using the parameters, partial density and MD, considering the correlation between the features most closely related to the performance. It evaluated feature combinations by considering the density and distance of overlapping regions specific to each class, enabling the selection of the most suitable features for the classification model in the rotating element diagnosis.

FPD algorithms can solve the problem of low accuracy, which is a limitation of existing filter-based feature-selection methods. As a result, the classifier based on the proposed algorithm provides a more accurate and time-efficient diagnosis of the rotating element in R2R systems than that achieved by other feature-selection methods. FPD, as a robust feature-selection algorithm, considers both density and distance based on classes, along with the most sensitive parameter for misclassification. It calculates PD in overlapping regions and quantifies the classification by considering the class distance in those regions.

## 6. Conclusions

Aiming to enhance efficient diagnosis of the operating status of rotational components in R2R production systems, this paper presents a feature-selection method based on partial density (FPD), which ultimately improves the coating quality and contributes to PHM. The FPD approach introduces the concept of partial density, which focuses solely on misclassified class data within overlapping regions. It also provides a quantitative evaluation method for classification by considering the ease of classification based on the Mahalanobis distance between the classes forming the partial density. Generally, a lower $FPD_{n}$ value indicates a higher classification accuracy, allowing for the ranking of feature combinations in ascending order based on $FPD_{n}$.

To validate the effectiveness of the proposed algorithm, a diagnostic experiment was conducted on the eccentric roll of an in-feeder roller within an industrial-scale R2R continuous production system. The top six and bottom six feature combinations were constructed based on the $FPD_{n}$ ranking of the collected vibration data, while an additional six feature combinations were randomly selected. The model trained using the top six feature combinations exhibited an average reduction in training time of 30.25% compared to that of the bottom six and random six feature combinations. Moreover, it demonstrated improvements of 37.90 in accuracy and 38.32% in the PPV, confirming the efficacy of the FPD algorithm-based feature selection. Furthermore, to highlight the superiority of the FPD method, feature combinations were selected using five previously studied feature-selection methods (mRMR, chi-square, ReliefF, MD evaluation, and FDM), and the training time, classification accuracy, and PPV were compared. The FPD method exhibited lower training times than classifiers employing mRMR, chi-square, ReliefF, MD evaluation, and FDM, by 44.17, 53.03, 56.01, 57.29, and 15.56%, respectively. Additionally, it achieved higher accuracies of 2.06, 8.45, 5.46, 11.53, and 0.83%, respectively, as well as higher PPVs of 1.67, 8.18, 5.49, 11.40, and 0.81%, respectively.

In conclusion, the proposed FPD algorithm effectively selects feature combinations for fault classification, reduces the training time of the rotational machine eccentricity diagnosis model in R2R systems and improves classification accuracy. This is achieved using a high-quality learning dataset to construct feature combinations that enhance accuracy and expedite training. The FPD algorithm accomplishes this by extracting class density by excluding the class with the maximum density and evaluating the classification rate based on the Mahalanobis distance between classes.

In this study, only SVM was used to verify the performance, and no other machine learning or deep learning techniques were used. In addition, since the data for the eccentricity experiment were collected in only one experimental setting, the data for various R2R system conditions [69,70,71] were not available, so it was not possible to verify the performance trend of the learning model according to the roll-to-roll system setup condition. Therefore, future research could use various machine learning and deep learning methods to achieve additional diagnostic performance from the technique and identify the impacts of different R2R system setup conditions like web materials, sensor types, imbalanced conditions, which could make significant contributions in computational domains and furthermore the physical domain.

Therefore, we plan to develop machine learning and deep learning-based diagnostic models for precise health diagnosis, prognosis, and health management (PHM) of R2R manufacturing systems and other manufacturing systems using unbalanced data collected from various sensors such as acceleration, vision, and tension sensors with various web materials such as metal and PET film.

## Figures and Tables

**Table 1:** Statistical feature variables used for feature extraction.

Statistical Feature	
Mean	Peak to peak
Median	Absolute mean
Mode	Crest factor
Trimmed mean	Skewness
RMS	Inverse of coefficient of variation
Standard deviation	K factor
Interquartile range	Kurtosis factor
Kurtosis	Fifth moment
Maximum	Sixth moment
Minimum	Clearance factor

**Table 2:** Specifications of the R2R system and accelerometer.

Item	Parameter	Value
R2R System	Web speed [mpm]	5
	Tension [kgf]	2.7
	Substrate	PET film (CD901, Kolon Inc., Seoul, Republic of Korea)
Sensor	Sensor type	Accelerometer
	Sensor model	356A01 and 356A15 (PCB Piezotronics, Depew, NY, USA)
	Sampling rate [kHz]	12.8
	Sampling duration [s]	60
	DAQ	NI-9230 and NI-9234 (National Instruments, Austin, TX, USA)
	DAQ module	NI-CDAQ-9174 (National Instruments, Austin, TX, USA)

**Table 3:** Classification result of feature combinations: kurtosis–peak to peak, median K factor, kurtosis-factor skewness.

FPD	Kurtosis–Peak to Peak	Median K Factor	Kurtosis-Factor Skewness
Parameter			
PD	0.206	0.199	0.365
MD	3.463	0.683	0.802
*FPD_n_*	0.059	0.292	0.455
Learning time [s]	1.705	1.975	2.243
Accuracy [%]	91.33	84.83	76.67
PPV [%]	93.06	91.47	82.52

**Table 4:** DNF number of each dataset in IFR-V data.

Sensor	1			2			3		
Axis	X	Y	Z	X	Y	Z	X	Y	Z
DNF_n_	1.091	1.104	1.094	1.101	1.126	1.103	1.080	1.085	1.082

**Table 5:** DNF number of each dataset in B-V data.

Housing	A		B	
Sensor direction	X	Y	X	Y
*DNF_n_*	6.321	14.334	1.816	2.154

**Table 6:** Classification results of the high-ranking six *FPD_n_* feature combinations.

FPD	Kurtosis–Peak to Peak	K Factor–Kurtosis	Kurtosis–6th Moment	Standard Deviation–Kurtosis	Maximum Kurtosis	Interquartile Range–Kurtosis
Parameter						
PD	0.206	0.159	0.137	0.142	0.183	0.195
MD	3.463	2.487	2.063	1.982	2.552	2.634
*FPD_n_*	0.059	0.064	0.066	0.071	0.072	0.074
Learning time [s]	1.705	1.724	1.976	1.968	1.940	1.767
Accuracy [%]	91.33	90.75	90.08	89.67	89.08	90.08
PPV [%]	93.06	93.27	92.57	92.05	91.65	91.25

**Table 7:** Classification results of the low-ranking six *FPD_n_* feature combinations.

FPD	Absolute Mean–Mean	Mean–Skewness	Skewness–Median	Skewness–Trimmed Mean	Skewness–Mode	Skewness–Absolute Mean
Parameter						
PD	0.428	0.468	0.470	0.469	0.464	0.466
MD	0.002	0.003	0.004	0.005	0.005	0.006
*FPD_n_*	241.020	144.158	120.185	103.373	88.827	83.794
Learning time [s]	3.008	2.518	2.691	2.620	2.612	2.436
Accuracy [%]	47.08	54.42	53.67	53.08	53.08	52.25
PPV [%]	46.24	58.10	55.79	55.10	55.04	53.64

**Table 8:** Classification results of six random feature combinations.

FPD	Absolute Mean–5th Moment	Median–K Factor	Kurtosis Factor–5th Moment	Interquartile Range–Peak to Peak	Trimmed Mean–6th Moment	Kurtosis–Factor Skewness
Parameter						
PD	0.350	0.199	0.309	0.216	0.168	0.365
MD	0.021	0.683	0.780	2.707	0.597	0.802
*FPD_n_*	16.398	0.292	0.396	0.080	0.280	0.455
Learning time [s]	2.478	1.878	2.334	1.886	2.588	2.473
Accuracy [%]	67.75	84.25	77.83	89.25	84.33	76.92
PPV [%]	78.71	90.53	84.65	91.24	92.92	83.44

**Table 9:** Comparison of average classification results of feature combination groups.

Parameter	High-Ranked Six	Low-Ranked Six	Random Six
*FPD_n_*	0.068	130.226	2.984
Learning time [s]	1.847	2.648	2.273
Accuracy [%]	90.17	52.26	80.06
PPV [%]	92.31	53.98	86.91

**Table 10:** Comparison of classification results according to the feature-selection method.

Parameter	mRMR	Chi-Square	ReliefF	MD Evaluation	FDM	*FPD_n_*
Learning time [s]	23.97	28.48	30.41	31.33	15.84	13.38
Accuracy [%]	93.15	86.75	89.75	83.67	94.37	95.21
PPV [%]	93.60	87.09	89.78	83.87	94.46	95.27

## Data Availability

The data (B-V data) presented in this study are openly available in https://doi.org/10.1016/J.DIB.2023.109049 (accessed on 26 July 2023) [48]. Data citation: Jung et al. 2023. Vibration, acoustic, temperature, and motor current dataset of rotating machine under varying operating conditions for fault diagnosis; Mendeley Data; Version 6; https://doi.org/10.17632/ztmf3m7h5x.6 (accessed on 26 July 2023) [72]. The other data (IFR-V data) presented in this study are available on request from the corresponding author.
